# Review of "Proteins of the Cerebrospinal Fluid" (2^nd ^Edition) by Edward J. Thompson

**DOI:** 10.1186/1743-8454-4-1

**Published:** 2007-01-26

**Authors:** James R Connor

**Affiliations:** 1 Department of Neurosurgery, Pennsylvania State University, M.S. Hershey Medical Center, Hershey PA 17033, USA

## Abstract

This book on cerebrospinal fluid (CSF) proteins is primarily focused on immunoglobulins. The book was written as an extension of a meeting on multiple sclerosis to provide a more extensive consideration of the CSF.

## Review

My review of this book by Dr. Thompson is written in the context of the reason I was drawn to this book and agreed to read the book and provide a review. I was drawn to the book based on its title because my research group was about to make a significant commitment to proteomic analyses of the cerebrospinal fluid (CSF) in both animal models and human disease states. I expected that, although I was familiar with CSF and the proteins that I had been studying for many years, this book would provide a foundation and reference for me and the postdoctoral fellows and students in my laboratory as we expanded our studies into the CSF. The forward indicated that the book "makes for an encyclopedia type of book for laboratory directors, experimentalists, etc". Therefore I was looking for a book on proteomics of the CSF and perhaps a state of the art assessment of protein profiling in the CSF. The first chapter of the book, a historical perspective on CSF proteins, mentions mostly IgG and associated methodologies. In Chapter 6 the discussion on pros and cons of qualitative versus quantitative analysis, a key issue in the evolving world of proteomics, is focused on IgG as the "central issue' in the studies of CSF proteins. In addition, the options for quantitative approaches do not provide much information on the strengths of ELISAs or even radioimmunoassays. The protocols are somewhat dated but in a historical context; a reasonable resource. Thus, this book is predominantly focused on immunoglobulins in the CSF with a particular focus on the oligoclonal IgG bands in multiple sclerosis. In the latter sense the book is an excellent resource and outstanding review of the field and the new investigator wishing to study immunoglobulin in the CSF can benefit from the comments of the author as a leader in this area from its beginning. As a resource itself, the book has 1,322 references. The reader interested in the broader topic of proteins in the CSF may find this book does not meet expectations.

The book is divided into four segments. Part 1 has 5 chapters and deals with normal CSF proteins in a "general fashion". This section is a strength of the book and provides useful background and organizational structure for the new student of the CSF. The tables are particularly helpful, providing information on the concentrations of different proteins in the CSF relative to serum. There is a very good reference table (4.4) on percentage transfer of proteins from serum to CSF. In contrast, many of the figures are difficult to understand at best, and are overly simplistic. For example, Figure 5.10 is a cartoon showing 6 sources of CSF but the 6 sources are not labeled or otherwise identified in a figure legend.

In some of the functional discussions in this section there are some questions about accuracy and some of the information is dated. Despite the abundance of references for this book, there are limited references in the discussion of the different blood and CSF barriers that should be provided to support some of the statements that invoke data as support. The function of prostaglandin synthase, induction of sleep when injected into the CSF, is confused with its end product PDG2. In another example, there is a statement that beta 2 microglobulin levels do not change in neurological disorders. Although this statement appears true for multiple sclerosis, there is certainly not uniform agreement on this in other neurological disorders [1-3]. Later, in Chapter 11, there is a comment stating that the ferritin synthesized within the brain is different from that found within the serum, but this has never been supported beyond the single paper that the author cites.

Another example of limited consideration of important topics is the rationale for why transferrin (Tf) without a sialic acid moiety (tau) occurs in the CSF. The treatment of this protein (and others) fails to consider the possibility that Tf made in the choroid plexus [4] can be released into the CSF and at one point refers to "presumed local synthesis" of Tf in Chapter 7. The figure provided as an explanation (Figure 5.7) for the presence of asialic Tf in the CSF is overly simplistic.

Comments such as protein released into the CSF by "white matter" may be carried down into the lumbar sac but any that are released through the cerebral cortex are swept into the arachnoid villi are difficult to understand and lack evidence to support them. Another such puzzling comment is that electron microscopists had previously assumed that there was no interstitial space until more recent techniques for tissue fixation were developed. Although historically accurate, the existence of interstitial space in the brain has been known for well over 30 years (see reference [5] for review).

Part 2 is described by the author as dealing with normal CSF proteins in a more specific fashion, but the chapters only review immunoglobulins. The subjects discussed can be, however, made applicable to other proteins. There is a discussion in Chapter 7 on movement of drugs across the BBB that will be appreciated by the novice investigator. Chapter 8 develops mathematical models to evaluate the source of IgG proteins in the CSF. Chapter 9 develops the idea that the most biologically stable values for normal barrier function are the ratios of IgG to albumin in CSF and serum. The rationale behind this latter statement is somewhat underdeveloped but does not appear to be inaccurate. The major focus of the chapter is again on the IgG levels. A number of points are introduced that could be extremely useful such as the observation that there is a circadian rhythm of the CSF, with a 6-fold increase in CSF production at night. This information is important for new investigators attempting to use CSF to obtain profiles for disease biomarkers and could have been emphasized more.

Part 3 is a "mini-clinical" book dealing with individual diseases. Chapter 10 of this section is dedicated to IgG production in the CSF, mostly in multiple sclerosis. The author misses an opportunity to use his historical perspective and experience in CSF biology to address the developing field of proteomics and the search for biomarkers for various diseases that reflect more than the degree of infection. In Chapter 11, the non-immunoglobulin proteins are given too little consideration and there are some bothersome comments such as myelin basic protein is referred to as the "best studied protein in the brain". Normal Pressure hydrocephalus is mentioned in the context of two proteins that appear enriched in the CSF of patients with this disorder. Given the vantage point of the author in CSF biology, this reader was left wanting for comments on the potential for using CSF protein profiles to differentiate NPH from Alzheimer's Disease. In context of the latter disease there is no mention of amyloid or A-beta fragments that many laboratories are trying to identify in CSF as a diagnostic indicator for AD [6]. The conclusion of Chapter 11 fails to mention proteomics when suggesting future directions for the use of CSF as a diagnostic marker. Chapter 13 is a reasonable review of the state of the field to use CSF as a prognostic indicator with a good summary table. In the chapter on future growth areas (Chapter 14) there are 5 lines dedicated to proteomics.

Part 4 is a mini laboratory book with a series of appendices providing protocols for analysis of CSF proteins. The protocols are standard gel analyses, but the author makes comments from his vast experience that would be helpful for those wishing to evaluate immunoglobulins in particular.

In summary, despite what I am sure appears to be a lukewarm review, this is not a disappointing book and is a good reference source for the new CSF investigator. The title is misleading and, thus if the reader is looking for a resource on CSF proteins and function, they will need to continue to look beyond this book. At the end of Chapter 14 on future growth areas the author concludes that "in the future, CSF is still not likely to be ignored". The author definitely reaches the right conclusion, but did not take full advantage of the opportunity to use this book and his considerable knowledge to serve as a guiding source for the next generation of CSF investigations and investigators.

## Competing interests

The author(s) declare that they have no competing interests.

## Authors' contributions

Sole author
